# Tubular secretion of creatinine and kidney function: an observational study

**DOI:** 10.1186/s12882-020-01736-6

**Published:** 2020-03-30

**Authors:** Xuehan Zhang, Andrew D. Rule, Charles E. McCulloch, John C. Lieske, Elaine Ku, Chi-yuan Hsu

**Affiliations:** 1 Department of Health Care, Peking Union Medical College Hospital, Chinese Academy of Medical Science & Peking Union Medical College, No. 1, Shuaifuyuan, Wangfujing St., Beijing, 100730 China; 2 Division of Nephrology and Hypertension, Mayo Clinic, Rochester, MN USA; 3 Division of Epidemiology, Mayo Clinic, Rochester, MN USA; 4 Department of Laboratory Medicine and Pathology, Mayo Clinic, Rochester, MN USA; 5 Division of Biostatistics, Department of Epidemiology and Biostatistics, University of California, San Francisco, San Francisco, CA USA; 6 Division of Nephrology, University of California, San Francisco, San Francisco, CA USA

**Keywords:** Chronic kidney disease (CKD), Glomerular filtration rate (GFR), Measurement error, Tubular secretion of creatinine, Creatinine clearance (CrCl)

## Abstract

**Background:**

Prior papers have been inconsistent regarding how much creatinine clearance (CrCl) overestimates glomerular filtration rate (GFR). A recent cross-sectional study suggested that measurement error alone could entirely account for the longstanding observation that CrCl/GFR ratio is larger when GFR is lower among patients with chronic kidney disease (CKD); but there have been no validation of this in other cohorts.

**Methods:**

To fill these gaps in knowledge regarding the relation between CrCl and GFR, we conducted cross-sectional and longitudinal analysis of the Modification of Diet in Renal Disease study (MDRD) and African American Study of Kidney Disease and Hypertension (AASK); and cross-sectional analysis of a clinical dataset from the Mayo Clinic of four different patient populations (CKD patients, kidney transplant recipients, post kidney donation subgroup and potential kidney donors). In the cross-sectional analyses (MDRD, AASK and Mayo Clinic cohort), we examined the relation between the CrCl/iothalamate GFR (iGFR) ratio at different categories of iGFR or different levels of CrCl. In the MDRD and AASK longitudinal analyses, we studied how the CrCl/iGFR ratio changed with those who had improvement in iGFR (CrCl) over time versus those who had worsening of iGFR (CrCl) over time.

**Results:**

Observed CrCl/iGFR ratios were generally on the lower end of the range reported in the literature for CKD (median 1.24 in MDRD, 1.13 in AASK and 1.25 in Mayo Clinic cohort). Among CKD patients in whom CrCl and iGFR were measured using different timed urine collections, CrCl/iGFR ratio were higher with lower iGFR categories but lower with lower CrCl categories. However, among CKD patients in whom CrCl and iGFR were measured using the same timed urine collections (which reduces dis-concordant measurement error), CrCl/iGFR ratio were higher with both lower iGFR categories and lower CrCl categories.

**Conclusions:**

These data refute the recent suggestion that measurement error alone could entirely account for the longstanding observation that CrCl/GFR ratio increases as GFR decreases in CKD patients. They also highlight the lack of certainty in our knowledge with regard to how much CrCl actually overestimates GFR.

## Background

Creatinine clearance (CrCl) has been used for decades as a proxy measure for glomerular filtration rate (GFR), and contemporary equations used to estimate GFR are based on serum creatinine concentration [1, 2]. Besides creatinine production and GFR, another factor which can influence serum creatinine concentration is tubular secretion (i.e. non-filtration clearance) of creatinine. But there are surprising gaps in knowledge regarding the relation between CrCl and GFR.

First, prior papers have been inconsistent regarding the degree to which CrCl overestimates GFR, with estimates ranging from around 10% [3–7] to over 60% [8, 9].

Second, we recently questioned whether the observed larger ratio of CrCl to measured GFR among those with lower GFR is actually due to proportionally greater tubular creatinine secretion with chronic kidney disease (CKD) progression, as is commonly believed, or whether this can be entirely accounted for by (random) measurement error [3, 10]. But our prior study was limited since it was based on only cross-sectional analysis of enrollees from one CKD study (Chronic Renal Insufficiency Cohort [CRIC]), and the CrCl and measured GFR were not obtained simultaneously [3].

To address these two issues, we sought to analyze datasets where a larger number of patients had undergone measurement of both CrCl and GFR. The Modification of Diet in Renal Disease study (MDRD) and African American Study of Kidney Disease and Hypertension (AASK) are ideal data sources since they are both rigorously conducted cohorts and national in scope. They have the additional advantage of containing repeated measured of both CrCl and GFR, thus allowing us to perform longitudinal analyses and add a new dimension not present in our prior paper. We also conducted cross-sectional analysis of 4 different Mayo Clinic patient cohorts that underwent clinically-indicated CrCl and GFR measurements that were performed simultaneously (which should reduce dis-concordant measurement error).

## Methods

### Study population

This study is based on information obtained from MDRD study participants, AASK study participants and a database of clinically obtained creatinine and iothalamate clearances performed at the Mayo Clinic, Rochester, Minnesota. De-identified data from the MDRD and AASK studies were obtained from the National Institutes of Diabetes and Digestive and Kidney Disease (NIDDK) Data Repository after Institutional Review Board (IRB) approval was obtained [11, 12]. The design and baseline characteristic of the MDRD Study and AASK Study have been previously described [13–18]. Briefly, in MDRD, 840 CKD patients aged 18–70 years with measured GFR 13–55 ml/min/1.73m^2^ were randomized to determine the effects of usual vs. low vs. very-low protein diet, and usual vs. low blood-pressure targets. In AASK, 1094 African-Americans aged 18–70 years with GFR 20–65 ml/min/1.73m^2^ were randomized to either strict versus usual BP control and different anti-hypertensive agents. Both MDRD and AASK study enrollees underwent repeated measures of urinary clearance of iothalamate to quantify GFR and repeated 24-h urine collections to quantify CrCl. To avoid the potential for regression to the mean, we chose to conduct our (cross-sectional and) longitudinal analyses in MDRD and AASK starting at the 12-month visit after randomization. After excluding enrollees who were missing iothalamate GFR (iGFR) or CrCl, 797 MDRD and 802 AASK participants remained with a measurement at the 12-month study visit. Furthermore, 680 MDRD and 688 AASK participants had a subsequent measurement of iGFR and CrCl at the 24-month study visit and they constituted the study population for longitudinal analysis.

The Mayo Clinic Renal Studies Unit database included patients who had a urinary iothalamate clearance (iGFR) with simultaneous timed urine creatinine from 2007 to 2012. The same urine collection (over a single timed period) was used to quantify iGFR and CrCl. De-identified data were obtained after appropriate IRB approval. For the present analysis, we excluded patients < 18 years of age, on dialysis, or with a history of urinary diversion. Also excluded were patients lacking a serum/plasma creatinine from the same day as iGFR, and patients with an iGFR or CrCl < 5 ml/min/1.73m^2^ or > 300 ml/min/1.73m^2^. Given expected differences in level of kidney function and other factors [19], we limited the current analysis to the four most common indications for iGFR testing: CKD patients (*n* = 1693), kidney transplant recipients (*n* = 1461), post kidney donation subgroup (*n* = 206) and potential kidney donors (*n* = 464) [19].

### CrCl and GFR measurements

In the MDRD study, GFR was measured as the urinary clearance of ^125^I-iothalamate (iGFR) after a subcutaneous injection of 35 microcuries without simultaneous administration of epinephrine. GFR was calculated as the ratio of time-weighted averages of urine excretion rates of the iothalamate marker and the serum concentrations of the marker over three to four consecutive collection periods [20]. The median intratest coefficient of variation (CV) for iGFR was 9.4% [20]. Serum and urine creatinine concentrations were measured by an alkaline picrate assay with Lloyd’s reagent and a kinetic alkaline picrate assay [21]. CrCl was calculated based on UV/P from a single 24-h urine collection and like iGFR, also standardized to body surface area.

In the AASK study, GFR was assessed by urinary clearance of ^125^I-iothalamate (iGFR) after a subcutaneous infection of 35 microcuries of ^125^I-iothalamate. GFR for each 30-min period was calculated using the logarithmic mean of the plasma ^125^I-iothalamate counts during the period. The arithmetic mean of the four collection periods was used to calculate the GFR. In 3.5% of the instances, GFR was calculated based on information from three collection periods [22–24]. The median CV for iGFR over the collection periods was 10.7% [22]. Serum and urine creatinine were measured using a kinetic alkaline picrate assay (Jaffe method) at the AASK Central Biochemistry Laboratory in the Department of Laboratory Medicine at Cleveland Clinic [23, 24]. CrCl was computed from creatinine excretion in a 24-h urine collection and a single measurement of serum creatinine [23], and like iGFR, also standardized to body surface area. In both MDRD and AASK cohorts, CrCl and iGFR were quantified using different timed urine collections.

In the Mayo Clinic cohort, GFR was measured by renal clearance of non-radiolabeled iothalamate [25]. The test was performed under a standardized protocol in which patients were fasted and underwent testing early in the day to minimize the effects of diet and diurnal variation on GFR. They were orally hydrated with 4–6 glasses of water prior to subcutaneous injection of non-radiolabeled iothalamate. Two hours later, renal clearance of iothalamate was measured over 45–60 min. Plasma and urine iothalamate concentrations were measured by liquid chromatography-tandem mass spectrometry (LC-MS/MS) [26]. Serum or plasma creatinine values were obtained using an isotope dilution mass spectrometry (IDMS)-traceable Roche enzymatic method (Roche P or D Modular or Roche Cobas C501, Indianapolis, IN) using a blood sample obtained within 24 h of the study. In contrast to CRIC, MDRD and AASK, in the Mayo Clinic participants, the same timed urine collection for each person was used to determine GFR and CrCl.

### Statistical analysis

In cross-sectional analyses (MDRD, AASK and Mayo Clinic cohort), we examined the relation between CrCl/iGFR ratio at different categories of iGFR or different levels of CrCl as in our prior approach [3]. We studied relative secretion of creatinine (CrCl/iGFR) because it is widely reported in the literature.

In the MDRD and AASK longitudinal analyses, mimicking the analysis by Shemesh et al. [8], we first divided the study population into those who had improvement in iGFR over time versus those who had worsening of iGFR over time. We then examined how the CrCl/iGFR ratio changed in these two groups. We subsequently divided the study population into those who had improvement in measured CrCl over time versus those who had worsening of measured CrCl over time to examine how the CrCl/iGFR ratio changed in these two groups.

We used mean and standard deviation or median and interquartile range as descriptive statistics to summarize distributions of parameters of interest. All statistical analyses were carried out using IBM SPSS Statistics 20.0 (IBM Corporation, Armonk, NY). Analysis of the Mayo data was independently confirmed using JMP software Version 10.0 (SAS Institute, Cary, NC).

## Results

### Cross-sectional analysis in MDRD and AASK

Among 797 MDRD participants, the median CrCl was 34 (interquartile range [IQR], 22–49) ml/min/1.73m^2^ and median iGFR 28 (IQR, 18–39) ml/min/1.73m^2^. Median CrCl/iGFR ratio was 1.24 (IQR, 1.10–1.37). Of 674 MDRD participants with related data, the median time lapse between 24-h urine collection and iGFR measurement was 0 day (IQR 0–1 day).

Among 802 AASK participants, the median CrCl was 48 (IQR, 31–66) ml/min/1.73m^2^ and median iGFR 46 (IQR, 33–59) ml/min/1.73m^2^. Median CrCl/iGFR ratio was 1.13 (IQR, 0.88–1.35).

**Fig. 1:**
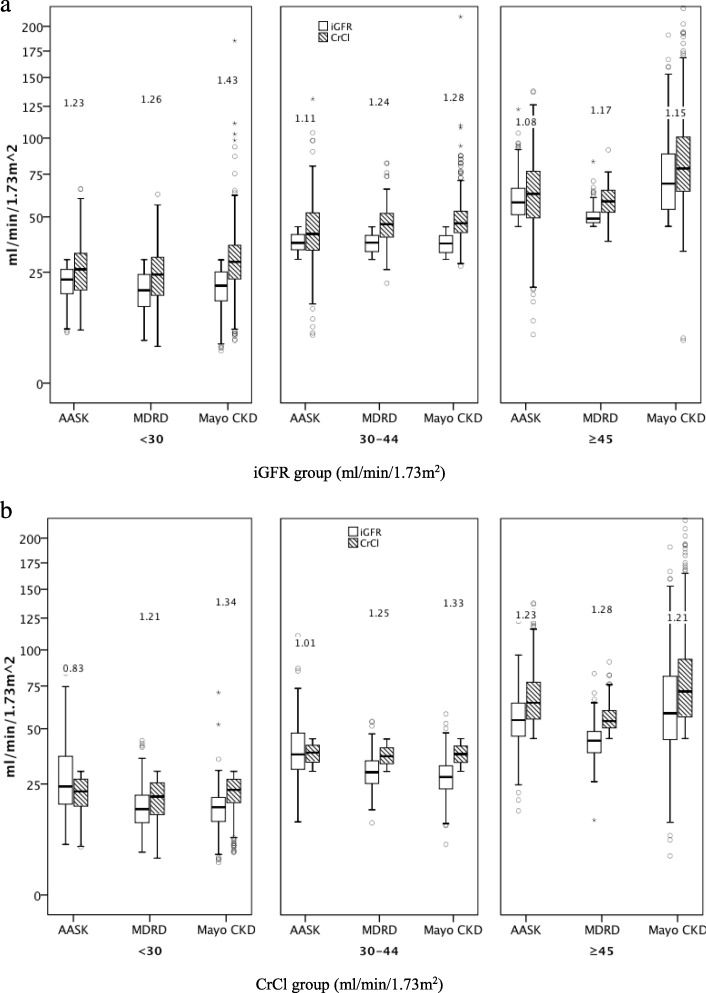
Distribution of iGFR, CrCl, as well as CrCl/iGFR ratio stratified by categories of iGFR (**a**) and by categories of CrCl (**b**) in 802 AASK, 797 MDRD and 1693 Mayo CKD participants (box plots show median, interquartile range and outliers; whiskers represent the highest and lowest values that are not outliers more than 1.5 box lengths from one hinge of the box). The data in the figure represents the corresponding median CrCl/iGFR ratios

We were able to replicate our prior CRIC cross-sectional findings [3] in MDRD and AASK. Specifically, the CrCl/iGFR ratio was progressively larger at lower iGFR level when patients were classified by categories of iGFR in both MDRD and AASK (Fig. 1a) (details in Additional files 1: Tables S1A and S2A). However, when the same patients were classified by categories of CrCl, the ratio of CrCl/iGFR was *smaller* at lower CrCl level (Fig. 1b) (details in Additional file 1: Tables S1B and S2B). Similar results were seen when tertiles of iGFR or CrCl were used for cutoff (data not shown) or in continuous analysis (Additional file 2: Figure S1 and S2).

### Longitudinal analysis in MDRD and AASK

**Fig. 2:**
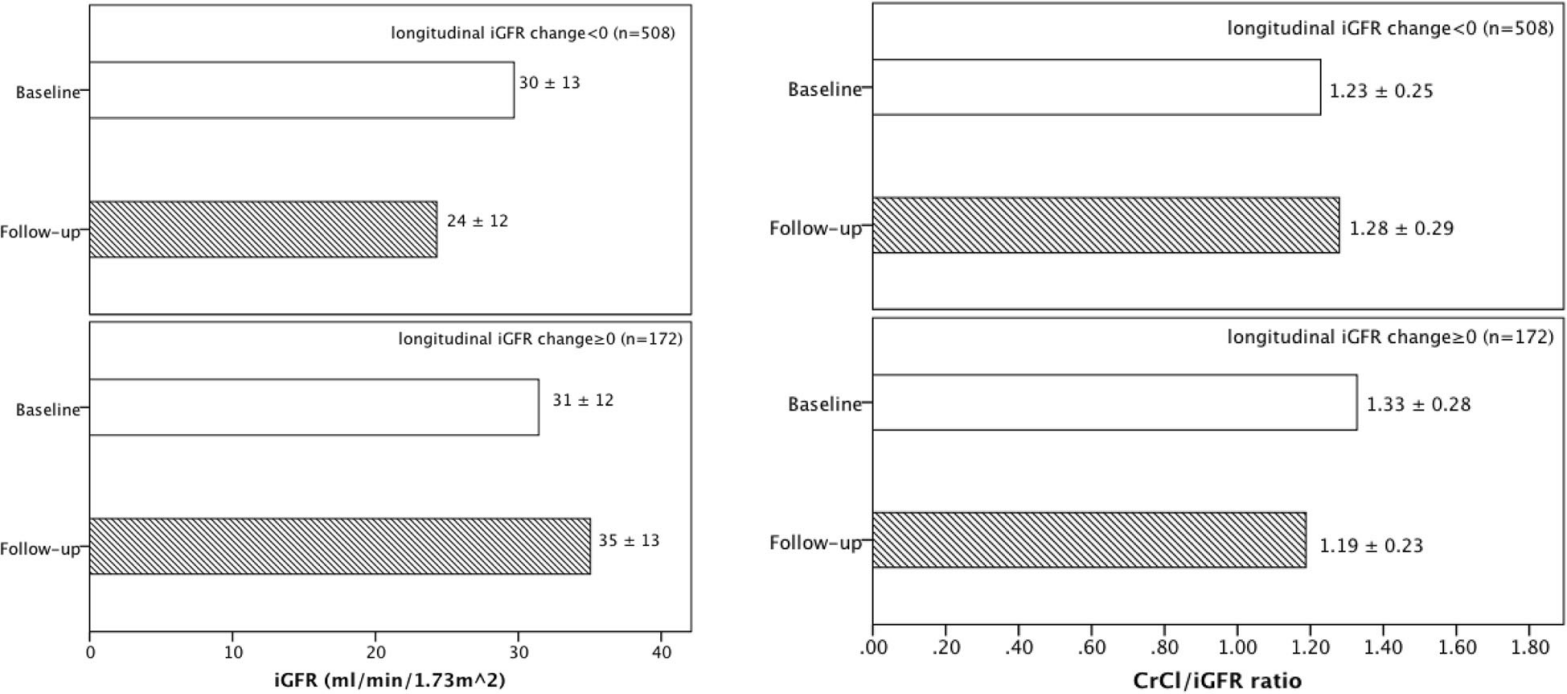
Change in mean (±standard deviation [SD]) iGFR and mean (±SD) CrCl/iGFR ratio longitudinally among MDRD study participants divided into those with decreasing iGFR (*n* = 508) or increasing iGFR (*n* = 172)

**Fig. 3:**
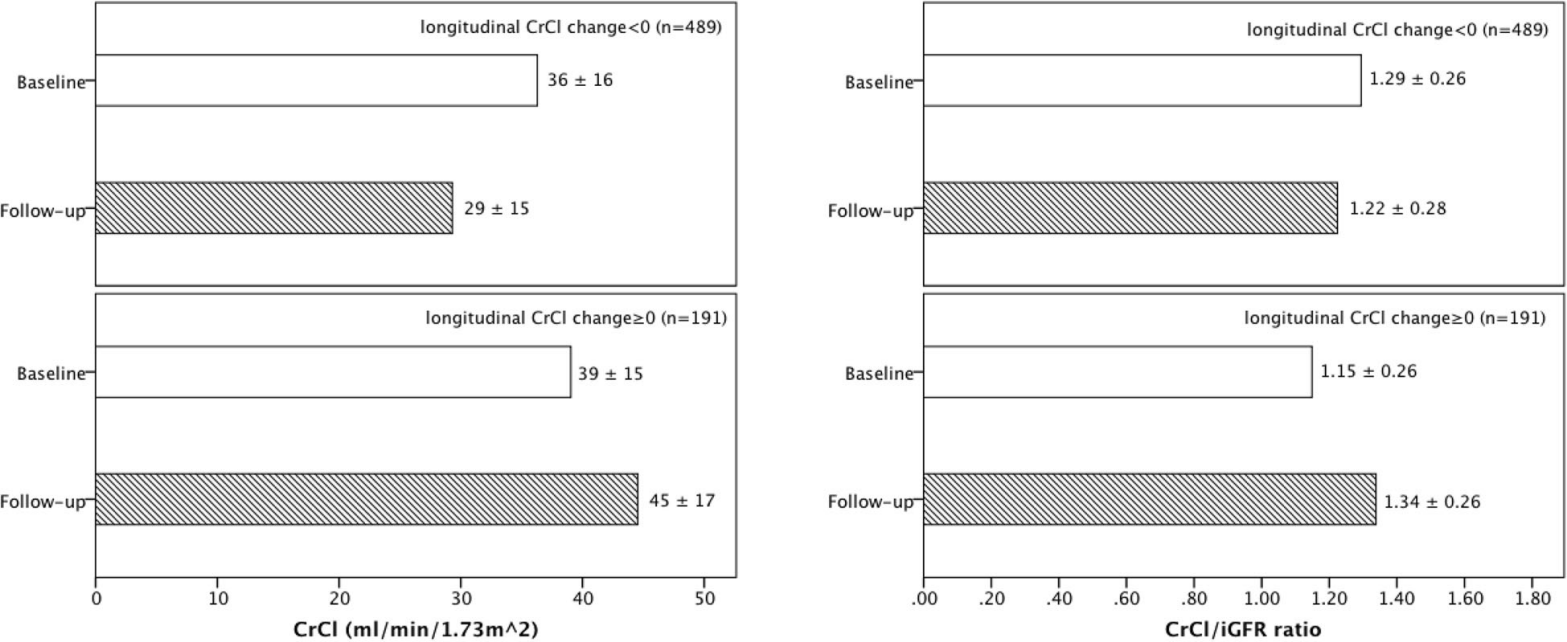
Change in mean (±SD) CrCl and mean (±SD) CrCl/iGFR ratio longitudinally among MDRD study participants divided into those with decreasing CrCl (*n* = 489) or increasing CrCl (*n* = 191)

In longitudinal analyses, median time gap between repeat assessment of kidney function was 12.0 (IQR, 8.3–12.3) months in 680 MDRD study participants. We looked at subsets who had improvement in iGFR (CrCl) over time versus those who had worsening of iGFR (CrCl) over time because we were interested in figuring out how the CrCl/iGFR ratio changed with iGFR (CrCl) changed over time. The CrCl/iGFR ratio increased among participants whose iGFR decreased over time, but CrCl/iGFR ratio decreased among those whose iGFR increased over time (Fig. 2); the opposite were observed with changes in CrCl (Fig. 3). These parallel findings in our cross-sectional analysis.

Similar results were observed in 688 AASK study participants (median time gap between repeat assessment of kidney function 12.2 (IQR, 11.7–17.1) months) (Additional file 3: Figure S3 and S4).

### Cross-sectional analysis in the Mayo Clinic subgroups: between-group comparison

Among the four Mayo Clinic subgroups, the potential kidney donors had the highest median iGFR (97 ml/min/1.73m^2^), followed by the post kidney donation subgroup (64 ml/min/1.73m^2^), the kidney transplant recipients (48 ml/min/1.73m^2^) and the CKD patients (41 ml/min/1.73m^2^) (Table 1).

**Table 1:** The distribution of iGFR, CrCl and CrCl/iGFR ratio in the 4 different subgroups of the Mayo Clinic cohort

Groups	iGFR (ml/min/1.73m^**2**^) Median (IQR)	CrCl (ml/min/1.73m^**2**^) Median (IQR)	CrCl/iGFR ratio Median (IQR)
Potential kidney donors (*n* = 464)	97 (85, 111)	102 (87, 116)	1.05 (0.96, 1.14)
Post kidney donation subgroup (n = 206)	64 (56, 73)	68 (61, 78)	1.09 (1.01, 1.19)
Kidney transplant recipients (n = 1461)	48 (34, 60)	58 (45, 72)	1.25 (1.09, 1.42)
CKD patients (n = 1693)	41 (25, 65)	52 (35, 77)	1.25 (1.11, 1.45)

The median CrCl/iGFR ratio was lowest in the potential kidney donors (1.05), followed by the post kidney donation subgroup (1.09), the kidney transplant recipients (1.25) and the CKD patients (1.25) (Table 1). Thus, in this *between group comparison*, lower kidney function—quantified *both* by iGFR and CrCl--was associated with a higher CrCl/iGFR ratio.

### Cross-sectional analysis in the Mayo Clinic subgroups: within group comparison

Within each of the 4 Mayo Clinic cohort subgroups, the CrCl/iGFR ratio in general were progressively *larger* with lower iGFR levels (Table 2) (details in Additional file 1: Table S3_1A, S3_2A, S3_3A and S3_4A).

**Table 2:** CrCl/iGFR ratio by categories of iGFR or CrCl (Mayo Clinic cohort subgroups)

CKD patients (n = 1693)	iGFR category (ml/min/1.73m^**2**^)								
				≥75 (*n* = 316)	60–74 (*n* = 168)	45–59 (*n* = 275)	30–44 (*n* = 355)	< 30 (*n* = 579)	*P*
	CrCl/iGFR ratio			1.08 (0.97, 1.21)	1.18 (1.06, 1.32)	1.21 (1.09, 1.32)	1.28 (1.14, 1.43)	1.43 (1.26, 1.68)	< 0.001
	CrCl category (ml/min/1.73m^**2**^)								
				≥75 (*n* = 453)	60–74 (*n* = 231)	45–59 (*n* = 321)	30–44 (*n* = 377)	< 30 (*n* = 311)	
	CrCl/iGFR ratio			1.18 (1.05, 1.36)	1.22 (1.06, 1.35)	1.23 (1.11, 1.40)	1.33 (1.16, 1.54)	1.34 (1.17, 1.55)	0.005
**Kidney transplant recipients** **(n = 1461)**	**iGFR category (ml/min/1.73m**^**2**^**)**								
				≥75 (*n* = 132)	60–74 (*n* = 239)	45–59 (*n* = 430)	30–44 (*n* = 410)	< 30 (*n* = 250)	
	CrCl/iGFR ratio			1.04 (0.91, 1.19)	1.14 (1.02, 1.29)	1.17 (1.06, 1.33)	1.31 (1.19, 1.46)	1.51 (1.35, 1.77)	< 0.001
	**CrCl category (ml/min/1.73m**^**2**^**)**								
				≥75 (*n* = 310)	60–74 (*n* = 341)	45–59 (*n* = 447)	30–44 (*n* = 263)	< 30 (*n* = 100)	
	CrCl/iGFR ratio			1.29 (1.12, 1.44)	1.19 (1.05, 1.36)	1.22 (1.06, 1.39)	1.27 (1.10, 1.50)	1.39 (1.18, 1.63)	< 0.001
**Post kidney donation subgroup (n = 206)**	**iGFR category (ml/min/1.73m**^**2**^**)**								
				≥75 (*n* = 42)	60–74 (*n* = 91)	< 60 (*n* = 73)			
	CrCl/iGFR ratio			1.06 (0.99, 1.13)	1.04 (0.97, 1.15)	1.14 (1.02, 1.31)			< 0.001
	**CrCl category (ml/min/1.73m**^**2**^**)**								
				≥75 (*n* = 75)	60–74 (*n* = 85)	< 60 (n = 46)			
	CrCl/iGFR ratio			1.15 (1.06, 1.22)	1.06 (1.00, 1.17)	1.02 (0.91, 1.12)			< 0.001
**Potential kidney donors** **(n = 464)**	**iGFR category (ml/min/1.73m**^**2**^**)**								
		≥110 (*n* = 123)	90–109 (*n* = 175)	< 90 (*n* = 166)					
	CrCl/iGFR ratio	0.98 (0.89, 1.07)	1.06 (0.97, 1.13)	1.09 (1.00, 1.19)					< 0.001
	**CrCl category (ml/min/1.73m**^**2**^**)**								
		≥110 (*n* = 159)	90–109 (*n* = 170)	< 90 (*n* = 135)					
	CrCl/iGFR ratio	1.10 (1.01, 1.20)	1.04 (0.96, 1.13)	0.99 (0.87, 1.08)					< 0.001

All the data are expressed as median (IQR)

When the participants were classified by categories of CrCl, the ratio of CrCl/iGFR were *smaller* with lower CrCl level in the Mayo Clinic potential kidney donors and post kidney donation subgroup (Table 2) (details in Additional file 1: Table S3_3B and S3_4B).

In the transplant recipients, there was not a simple stepwise relation between CrCl/iGFR ratio and categories of CrCl, but below CrCl of 75 ml/min/1.73m^2^ the ratio of CrCl/iGFR were progressively *larger* with lower CrCl level (Table 2) (details in Additional file 1: Table S3_2B).

However, among the Mayo Clinic CKD patients--in contrast to what was observed in the CKD populations of CRIC, MDRD and AASK--the ratio of CrCl/iGFR were progressively *larger* with lower CrCl level (Table 2, Fig. 1b) (details in Additional file 1: Table S3_1B).

## Discussion

Although it has been known for many years that creatinine is cleared via both filtration and secretion, basic gaps remain in our knowledge regarding the physiology and pathophysiology of tubular secretion of creatinine in various states of health and disease. For example, exactly what fraction of creatinine is cleared via secretion vs. filtration? Is it possible that measurement error alone could entirely account for the longstanding observation that CrCl/GFR ratio is larger at lower GFR among patients with CKD? These questions are pertinent in the context of renewed recent interest in renal tubular function [27–29]. Better understanding of tubular secretion as an independent marker of kidney function may provide insight into kidney disease pathophysiology and improve prediction of adverse outcomes [27]. Others have emphasized that many drugs are cleared by tubular secretion, and drug-dose modification in CKD should not assume that renal excretory processes always decline in parallel with GFR as CKD progresses [30].

In this study we showed the following. First, we are able to replicate the results of our original CRIC cross-sectional results in MDRD and AASK [3]. Replicating our initial findings in two other large research cohorts underscores the idea that certain studies in the literature may have been biased by measurement error, which has been under-appreciated in the past. (In fact, measurement error has not been mentioned at all in the prior literature as far as we can tell.)

Second (and closely related conceptually), we demonstrate that measurement error likely played an under-appreciated role also in prior literature that describes longitudinal changes in the CrCl/GFR ratio. For example, in the much cited study by Shemesh et al. [8], the authors concluded from their longitudinal analysis that “The opposite changes in fractional creatinine secretion and GFR as glomerular disease deteriorates or improves serve to blunt the magnitude of change when creatinine is used to monitor progression of the glomerular injury [8].” Not considered by the authors is the fact that measurement error alone may explain much of this observation.

Third, since inaccurate collection of urine specimens is the biggest source of measurement error for directly measured GFR and CrCl by urinary clearance, when both CrCl and GFR were measured using the same timed urine collections, the role of measurement error should be considerably reduced. In this context, the findings in the Mayo Clinic cohort CKD patients (and kidney transplant recipients with CrCl < 75 ml/min/1.73 m^2^) are of particular interest. In these cohorts that used the same urine collection to quantify iGFR and CrCl we no longer observed a decreasing CrCl/iGFR ratio at progressively lower CrCl. Instead, in these patients the CrCl/iGFR ratio goes up in cross-sectional analysis with progressively lower CrCl (and progressively lower iGFR). This—and the between-group comparisons shown in Table 1---supports the textbook teaching that there is indeed proportionally greater tubular creatinine secretion with worsening kidney function (since those with lower CrCl have worse kidney function). These data demonstrate that measurement error alone could not entirely account for the longstanding observation that CrCl/GFR ratio increases as GFR decreases in patients with CKD.

Fourth, we note that there is considerable variation in the average CrCl/iGFR ratio across studies. In CRIC study, the median ratio was 1.09 [3]. In MDRD study it was 1.24 and in AASK study it was 1.13. One problem is that these and other older studies [3–9] were done prior to the era of standardization of the IDMS-traceable reference calibrator. Another problem is that different studies used different methods to measure GFR and these are known to be inconsistent with each other [31–33], thus rendering definitive conclusions difficult. Even within our study, serum creatinine was measured using the enzymatic method at the Mayo Clinic but not in MDRD or AASK. The alkaline picrate method for the measurement of creatinine can be influenced by non-creatinine chromogens (such as acetoacetate and some antibiotics), especially on older platforms [34]. The so-called ‘Jaffe’ method has no standard recipe and much methodological variation has occurred over time [35]. Interestingly in the Mayo Clinic data (Table 2), the CrCl/iGFR ratio appears to differ by type of clinical presentation. For example, for the category of iGFR 60–74 ml/min/1.73m^2^, median CrCl/iGFR ratio was 1.18 in CKD patients but 1.04 in the post kidney donation subgroup. So perhaps it appears that despite decades of research and seemingly definitive statements in textbooks and review articles, we in fact do not know with certitude the degree by which CrCl overestimates GFR.

Strengths of our study include validation of our prior findings (in CRIC) [3] from two other large CKD research studies (MDRD and AASK). We extended our cross-sectional analysis to longitudinal analyses to bring attention to the fact that measurement error could also have contributed to prior report of temporal changes in CrCl/GFR over time within a person [8]. Finally, the Mayo Clinic data, based on using the same timed urine collection to measure CrCl and GFR, shed new light on the limited role of measurement error.

We also recognize several limitations. First, we did not measure iGFR and CrCl using the same blood samples in the Mayo Clinic patients which would have even further reduced measurement error. But previous Mayo Clinic studies have confirmed that a routine fasting morning serum creatinine drawn within 24-h of the study and a plasma creatinine obtained during the iothalamate study do not significantly differ (data not shown). Second, we did not have information on the concomitant use of drugs, such as trimethoprim or cimetidine, which could have affected tubular secretion of creatinine. Third, we did not perform interventional studies with agents such as cimetidine to examine the effect of blocking tubular secretion of Cr [36–39]. However, these experiments are not straightforward since cimetidine is cleared by the kidneys, so in patients with more advanced CKD, less cimetidine is filtered and more cimetidine becomes available in the proximal tubular pericapillary circulation. Thus, more cimetidine enters the proximal tubular cells to compete with creatinine for the brush border (luminal) secretory transporter [39]. It is also not clear how complete inhibition can be established. Interestingly, one older study which attempted to do that reported that “amount of overestimation of true GFR by creatinine clearance was not associated with extent of renal functional impairment [37].” However, other studies suggested that the tubular secretion of cimetidine increases inversely with GFR [36, 38, 39].

## Conclusions

To close, our prior study highlighted measurement error as alternative interpretation of the literature which has hitherto not been considered [3]. The current study demonstrates that measurement error alone could not entirely account for the longstanding observation that CrCl/GFR ratio increases as GFR decreases in patients with CKD. Our data also draw attention to gaps in knowledge with regard to how much CrCl actually overestimates GFR, despite decades of literature on this topic.

## Supplementary information

**Additional file 1 Table S1**. CrCl/iGFR classified by categories based on iGFR and CrCl (MDRD data). **Table S2**. CrCl/iGFR classified by categories based on iGFR and CrCl (AASK data). **Table S3_1**. CrCl/iGFR classified by categories based on iGFR and CrCl (Mayo data, CKD patients). **Table S3_2**. CrCl/iGFR classified by categories based on iGFR and CrCl (Mayo data, kidney transplant recipients). **Table S3_3**. CrCl/iGFR classified by categories based on iGFR and CrCl (Mayo data, post kidney donation subgroup). **Table S3_4**. CrCl/iGFR classified by categories based on iGFR and CrCl (Mayo data, potential kidney donors).

**Additional file 2 Fig. S1**. (A) Relationship between CrCl/iGFR ratio and iGFR, (B) Relationship between CrCl/iGFR ratio and CrCl (The line shown through the scatterplot is from LOESS) (MDRD data, *n* = 797). **Fig. S2**. (A) Relationship between CrCl/iGFR ratio and iGFR, (B) Relationship between CrCl/iGFR ratio and CrCl (The line shown through the scatterplot is from LOESS) (AASK data, *n* = 802).

**Additional file 3 Fig. S3**. Change in mean (±SD) iGFR and mean (±SD) CrCl/iGFR ratio longitudinally among AASK study participants divided into those with decreasing iGFR or increasing iGFR. **Fig. S4**. Change in mean (±SD) CrCl and mean (±SD) CrCl/iGFR ratio longitudinally among AASK study participants divided into those with decreasing CrCl or increasing CrCl.

## Data Availability

The data from the MDRD and the AASK studies reported here are available upon request in the NIDDK Central Repositories. The data from the Mayo Clinic Renal Studies Unit database are not publicly available due to the confidential nature of patient information obtained for clinical care.

## Abbreviations

AASK: African American Study of Kidney Disease and Hypertension
CKD: Chronic kidney disease
CrCl: Creatinine clearance
CRIC: Chronic Renal Insufficiency Cohort
CV: Coefficient of variation
GFR: Glomerular filtration rate
iGFR: Glomerular filtration rate by iothalamate clearance
MDRD: Modification of Diet in Renal Disease
