# Supplementary material for: Tubular secretion of creatinine and kidney function: an observational study
Source: BMC Nephrol. 2020 Mar 30;21:108. doi: 10.1186/s12882-020-01736-6 (PMC7104490; doi:10.1186/s12882-020-01736-6)
Supplement: Supplementary file 1 — Additional file 1 Table S1. CrCl/iGFR classified by categories based on iGFR and CrCl (MDRD data). Table S2. CrCl/iGFR classified by categories based on iGFR and CrCl (AASK data). Table S3_1. CrCl/iGFR classified by categories based on iGFR and CrCl (Mayo data, CKD patients). Table S3_2. CrCl/iGFR classified by categories based on iGFR and CrCl (Mayo data, kidney transplant recipients). Table S3_3. CrCl/iGFR classified by categories based on iGFR and CrCl (Mayo data, post kidney donation subgroup). Table S3_4. CrCl/iGFR classified by categories based on iGFR and CrCl (Mayo data, potential kidney donors). [file 12882_2020_1736_MOESM1_ESM.docx]

**Table S1. CrCl/iGFR classified by categories based on iGFR (A) and CrCl (B) (MDRD data, n=797)**

| **(A)** |  |  |  |  | |
| --- | --- | --- | --- | --- | --- |
| **iGFR level (ml/min/1.73m2)** | **iGFR (ml/min/1.73m2)**  median (5th, 25th, 75th , 95th) | **CrCl (ml/min/1.73m2)**  median (5th, 25th, 75th , 95th) | **CrCl/iGFR**  median (5th, 25th, 75th , 95th) | | **CrCl/iGFR**  mean ± SD |
| ≥45 (n=116) | 49  (45, 47, 53, 63) | 58  (44, 52, 65, 75) | 1.17  (0.83, 1.06, 1.28, 1.44) | 1.16 ± 0.18 | |
| 30-44 (n=235) | 37  (31, 33, 41, 44) | 46  (31, 40, 52, 60) | 1.24  (0.87, 1.08, 1.38, 1.59) | 1.24 ± 0.24 | |
| <30 (n=446) | 19  (9, 14, 24, 29) | 24  (10, 17, 31, 39) | 1.26  (0.86, 1.12, 1.41, 1.79) | 1.29 ± 0.30 | |
| **(B)** |  |  |  |  | |
| **CrCl level**  **(ml/min/1.73m2)** | **iGFR (ml/min/1.73m2)**  **median (5th, 25th, 75th , 95th)** | **CrCl (ml/min/1.73m2)**  **median (5th, 25th, 75th , 95th)** | **CrCl/iGFR**  **median (5th, 25th, 75th , 95th)** | **CrCl/iGFR**  **mean ± SD** | |
| ≥45 (n=241) | 44  (31, 38, 49, 60) | 54  (46, 50, 60, 74) | 1.28  (1.02, 1.16, 1.41, 1.78) | 1.31 ± 0.27 | |
| 30-44 (n=223) | 30  (21, 25, 34, 44) | 37  (31, 33, 41, 44) | 1.25  (0.88, 1.08, 1.37, 1.72) | 1.26 ± 0.26 | |
| <30 (n=333) | 16  (8, 12, 21, 29) | 20  (9, 15, 25, 29) | 1.21  (0.77, 1.04, 1.36, 1.69) | 1.21 ± 0.28 | |

**Table S2. CrCl/iGFR classified by categories based on iGFR (A) and CrCl (B) (AASK data, n=802)**

| **(A)** |  |  |  |  |
| --- | --- | --- | --- | --- |
| **iGFR level (ml/min/1.73m^2^)** | **iGFR (ml/min/1.73m^2^)**  median (5^th^, 25^th^, 75^th^ , 95^th^) | **CrCl (ml/min/1.73m^2^)**  median (5^th^, 25^th^, 75^th^ , 95^th^) | **CrCl/iGFR**  median (5^th^, 25^th^, 75^th^ , 95^th^) | **CrCl/iGFR**  mean ± SD |
| ≥60 (n=174) | 68  (61, 63, 74, 92) | 72  (33, 56, 85, 111) | 1.02  (0.47, 0.81, 1.21, 1.53) | 1.02 ± 0.32 |
| 45-59 (n=240) | 52  (45, 49, 56, 59) | 58  (25, 44, 70, 88) | 1.12  (0.47, 0.86, 1.34, 1.64) | 1.10 ± 0.36 |
| 30-44 (n=217) | 37  (31, 34, 41, 44) | 41  (18, 34, 52, 68) | 1.11  (0.47, 0.89, 1.35, 1.79) | 1.15 ± 0.43 |
| <30 (n=171) | 22  (12, 18, 26, 29) | 26  (11, 19, 33, 46) | 1.23  (0.54, 1.00, 1.56, 2.02) | 1.28 ± 0.46 |
| **(B)** |  |  |  |  |
| **CrCl level**  **(ml/min/1.73m^2^)** | **iGFR (ml/min/1.73m^2^)**  median (5^th^, 25^th^, 75^th^ , 95^th^) | **CrCl (ml/min/1.73m^2^)**  median (5^th^, 25^th^, 75^th^ , 95^th^) | **CrCl/iGFR**  median (5^th^, 25^th^, 75^th^ , 95^th^) | **CrCl/iGFR**  mean ± SD |
| ≥60 (n=263) | 59  (41, 51, 67, 85) | 73  (61, 66, 85, 105) | 1.29  (0.89, 1.12, 1.47, 1.93) | 1.33 ± 0.35 |
| 45-59 (n=159) | 47  (29, 39, 57, 71) | 53  (46, 49, 56, 59) | 1.12  (0.74, 0.95, 1.35, 1.77) | 1.18 ± 0.35 |
| 30-44 (n=197) | 37  (23, 31, 48, 66) | 38  (31, 34, 42, 44) | 1.01  (0.54, 0.82, 1.23, 1.70) | 1.05 ± 0.36 |
| <30 (n=183) | 24  (13, 18, 37, 57) | 22  (9, 17, 27, 29) | 0.83  (0.32, 0.53, 1.20, 1.65) | 0.89 ± 0.41 |

**Table S3_1. CrCl/iGFR classified by categories based on iGFR (A) and CrCl (B) (Mayo data, CKD patients, n=1693)**

| **(A)** |  |  | |  |  |
| --- | --- | --- | --- | --- | --- |
| **iGFR level (ml/min/1.73m^2^)** | **iGFR (ml/min/1.73m^2^)**  median (5^th^, 25^th^, 75^th^ , 95^th^) | **CrCl (ml/min/1.73m^2^)**  median (5^th^, 25^th^, 75^th^ , 95^th^) | **CrCl/iGFR**  median (5^th^, 25^th^, 75^th^ , 95^th^) | | **CrCl/iGFR**  mean ± SD |
| ≥75 (n=316) | 93  (77, 84, 107, 128) | 102  (72, 89, 117, 159) | 1.08  (0.82, 0.97, 1.21, 1.48) | | 1.10 ± 0.21 |
| 60-74 (n=168) | 67  (60, 64, 71, 74) | 78  (60, 70, 86, 112) | 1.18  (0.89, 1.06, 1.32, 1.60) | | 1.22 ± 0.31 |
| 45-59 (n=275) | 51  (46, 48, 55, 59) | 62  (46, 56, 68, 84) | 1.21  (0.91, 1.09, 1.32, 1.58) | | 1.21 ± 0.23 |
| 30-44 (n=355) | 37  (31, 33, 41, 44) | 47  (35, 42, 53, 73) | 1.28  (1.00, 1.14, 1.43, 1.94) | | 1.34 ± 0.35 |
| <30 (n=579) | 20  (8, 15, 25, 29) | 29  (13, 22, 36, 48) | 1.43  (1.07, 1.26, 1.68, 2.44) | | 1.58 ± 0.77 |
| **(B)** |  |  | |  |  |
| **CrCl level**  **(ml/min/1.73m^2^)** | **iGFR (ml/min/1.73m^2^)**  median (5^th^, 25^th^, 75^th^ , 95^th^) | **CrCl (ml/min/1.73m^2^)**  median (5^th^, 25^th^, 75^th^ , 95^th^) | **CrCl/iGFR**  median (5^th^, 25^th^, 75^th^ , 95^th^) | | **CrCl/iGFR**  mean ± SD |
| ≥75 (n=453) | 84  (44, 69, 98, 126) | 97  (77, 85, 111, 158) | 1.18  (0.90, 1.05, 1.36, 2.09) | | 1.32 ± 0.70 |
| 60-74 (n=231) | 56  (38, 49, 64, 80) | 67  (61, 63, 71, 74) | 1.22  (0.85, 1.06, 1.35, 1.71) | | 1.27 ± 0.56 |
| 45-59 (n=321) | 42  (27, 35, 48, 57) | 52  (45, 48, 56, 59) | 1.23  (0.95, 1.11, 1.40, 1.93) | | 1.31 ± 0.53 |
| 30-44 (n=377) | 28  (17, 23, 32, 42) | 38  (31, 34, 41, 44) | 1.33  (0.97, 1.16, 1.54, 2.12) | | 1.41 ± 0.40 |
| <30 (n=311) | 17  (7, 13, 20, 26) | 23  (9, 18, 27, 29) | 1.34  (0.91, 1.17, 1.55, 2.04) | | 1.39 ± 0.36 |

**Table S3_2. CrCl/iGFR classified by categories based on iGFR (A) and CrCl (B) (Mayo data, kidney transplant recipients, n=1461)**

| **(A)** |  |  |  |  |
| --- | --- | --- | --- | --- |
| **iGFR level (ml/min/1.73m^2^)** | **iGFR (ml/min/1.73m^2^)**  median (5^th^, 25^th^, 75^th^ , 95^th^) | **CrCl (ml/min/1.73m^2^)**  median (5^th^, 25^th^, 75^th^ , 95^th^) | **CrCl/iGFR**  median (5^th^, 25^th^, 75^th^ , 95^th^) | **CrCl/iGFR**  mean ± SD |
| ≥75 (n=132) | 81  (75, 78, 87, 100) | 86  (63, 75, 99, 144) | 1.04  (0.79, 0.91, 1.19, 1.51) | 1.08 ± 0.27 |
| 60-74 (n=239) | 66  (61, 63, 70, 74) | 76  (58, 67, 85, 105) | 1.14  (0.87, 1.02, 1.29, 1.59) | 1.17 ± 0.24 |
| 45-59 (n=430) | 53  (46, 49, 56, 59) | 62  (46, 55, 70, 82) | 1.17  (0.90, 1.06, 1.33, 1.60) | 1.21 ± 0.24 |
| 30-44 (n=410) | 38  (31, 34, 42, 44) | 49  (37, 43, 56, 69) | 1.31  (1.00, 1.19, 1.46, 1.82) | 1.35 ± 0.29 |
| <30 (n=250) | 22  (10, 18, 26, 29) | 33  (16, 27, 39, 53) | 1.51  (1.11, 1.35, 1.77, 2.33) | 1.60 ± 0.47 |
| **(B)** |  |  |  |  |
| **CrCl level**  **(ml/min/1.73m^2^)** | **iGFR (ml/min/1.73m^2^)**  median (5^th^, 25^th^, 75^th^ , 95^th^) | **CrCl (ml/min/1.73m^2^)**  median (5^th^, 25^th^, 75^th^ , 95^th^) | **CrCl/iGFR**  median (5^th^, 25^th^, 75^th^ , 95^th^) | **CrCl/iGFR**  mean ± SD |
| ≥75 (n=310) | 68  (46, 60, 78, 95) | 85  (76, 79, 94, 121) | 1.29  (0.96, 1.12, 1.44, 1.98) | 1.36 ± 0.42 |
| 60-74 (n=341) | 56  (38, 50, 64, 77) | 66  (61, 63, 71, 74) | 1.19  (0.88, 1.05, 1.36, 1.72) | 1.24 ± 0.36 |
| 45-59 (n=447) | 43  (30, 37, 50, 61) | 53  (46, 49, 56, 59) | 1.22  (0.88, 1.06, 1.39, 1.75) | 1.25 ± 0.27 |
| 30-44 (n=263) | 30  (18, 25, 36, 47) | 39  (31, 36, 42, 44) | 1.27  (0.90, 1.10, 1.50, 1.96) | 1.33 ± 0.33 |
| <30 (n=100) | 18  (8, 14, 21, 29) | 25  (13, 20, 28, 30) | 1.39  (0.94, 1.18, 1.63, 2.21) | 1.44 ± 0.39 |

**Table S3_3. CrCl/iGFR classified by categories based on iGFR (A) and CrCl (B) (Mayo data, post kidney donation subgroup, n=206)**

| **(A)** |  |  |  |  |
| --- | --- | --- | --- | --- |
| **iGFR level (ml/min/1.73m^2^)** | **iGFR (ml/min/1.73m^2^)**  median (5^th^, 25^th^, 75^th^ , 95^th^) | **CrCl (ml/min/1.73m^2^)**  median (5^th^, 25^th^, 75^th^ , 95^th^) | **CrCl/iGFR**  median (5^th^, 25^th^, 75^th^ , 95^th^) | **CrCl/iGFR**  mean ± SD |
| ≥75 (n=42) | 83  (75, 79, 87, 96) | 86  (68, 79, 97, 128) | 1.06  (0.84, 0.99, 1.13, 1.39) | 1.07 ± 0.16 |
| 60-74 (n=91) | 67  (61, 63, 70, 74) | 71  (54, 65, 77, 86) | 1.04  (0.82, 0.97, 1.15, 1.25) | 1.06 ± 0.14 |
| <60 (n=73) | 52  (38, 48, 57, 60) | 60  (45, 53, 66, 85) | 1.14  (0.92, 1.02, 1.31, 1.62) | 1.19 ± 0.22 |
| **(B)** |  |  |  |  |
| **CrCl level**  **(ml/min/1.73m^2^)** | **iGFR (ml/min/1.73m^2^)**  median (5^th^, 25^th^, 75^th^ , 95^th^) | **CrCl (ml/min/1.73m^2^)**  median (5^th^, 25^th^, 75^th^ , 95^th^) | **CrCl/iGFR**  median (5^th^, 25^th^, 75^th^ , 95^th^) | **CrCl/iGFR**  mean ± SD |
| ≥75 (n=75) | 75  (54, 67, 83, 91) | 83  (76, 78, 92, 114) | 1.15  (0.98, 1.06, 1.22, 1.68) | 1.18 ± 0.21 |
| 60-74 (n=85) | 62  (47, 57, 69, 81) | 67  (60, 63, 70, 74) | 1.06  (0.86, 1.00, 1.17, 1.37) | 1.09 ± 0.15 |
| <60 (n=46) | 53  (35, 48, 61, 69) | 54  (41, 49, 57, 60) | 1.02  (0.78, 0.91, 1.12, 1.35) | 1.02 ± 0.16 |

**Table S3_4. CrCl/iGFR classified by categories based on iGFR (A) and CrCl (B) (Mayo data, potential kidney donors, n=464)**

| **(A)** |  |  |  |  |
| --- | --- | --- | --- | --- |
| **iGFR level (ml/min/1.73m^2^)** | **iGFR (ml/min/1.73m^2^)**  median (5^th^, 25^th^, 75^th^ , 95^th^) | **CrCl (ml/min/1.73m^2^)**  median (5^th^, 25^th^, 75^th^ , 95^th^) | **CrCl/iGFR**  median (5^th^, 25^th^, 75^th^ , 95^th^) | **CrCl/iGFR**  mean ± SD |
| ≥110 (n=123) | 121  (111, 116, 127, 144) | 119  (89, 107, 131, 160) | 0.98  (0.72, 0.89, 1.07, 1.29) | 0.97 ± 0.16 |
| 90-109 (n=175) | 99  (91, 95, 105, 109) | 104  (80, 95, 115, 130) | 1.06  (0.80, 0.97, 1.13, 1.32) | 1.05 ± 0.15 |
| <90 (n=166) | 81  (60, 74, 86, 90) | 86  (60, 78, 97, 113) | 1.09  (0.87, 1.00, 1.19, 1.44) | 1.12 ± 0.20 |
| **(B)** |  |  |  |  |
| **CrCl level**  **(ml/min/1.73m^2^)** | **iGFR (ml/min/1.73m^2^)**  median (5^th^, 25^th^, 75^th^ , 95^th^) | **CrCl (ml/min/1.73m^2^)**  median (5^th^, 25^th^, 75^th^ , 95^th^) | **CrCl/iGFR**  median (5^th^, 25^th^, 75^th^ , 95^th^) | **CrCl/iGFR**  mean ± SD |
| ≥110 (n=159) | 113  (84, 101, 123, 140) | 121  (111, 116, 131, 156) | 1.10  (0.92, 1.01, 1.20, 1.45) | 1.14 ± 0.20 |
| 90-109 (n=170) | 96  (80, 89, 105, 129) | 100  (91, 96, 105, 108) | 1.04  (0.81, 0.96, 1.13, 1.26) | 1.04 ± 0.13 |
| <90 (n=135) | 81  (58, 74, 90, 112) | 81  (56, 74, 86, 89) | 0.99  (0.74, 0.87, 1.08, 1.22) | 0.98 ± 0.16 |
