# Supplementary material for: Tubular secretion of creatinine and kidney function: an observational study
Source: BMC Nephrol. 2020 Mar 30;21:108. doi: 10.1186/s12882-020-01736-6 (PMC7104490; doi:10.1186/s12882-020-01736-6)
Supplement: Supplementary file 2 — Additional file 2 Fig. S1. (A) Relationship between CrCl/iGFR ratio and iGFR, (B) Relationship between CrCl/iGFR ratio and CrCl (The line shown through the scatterplot is from LOESS) (MDRD data, n = 797). Fig. S2. (A) Relationship between CrCl/iGFR ratio and iGFR, (B) Relationship between CrCl/iGFR ratio and CrCl (The line shown through the scatterplot is from LOESS) (AASK data, n = 802). [file 12882_2020_1736_MOESM2_ESM.docx]

| A.  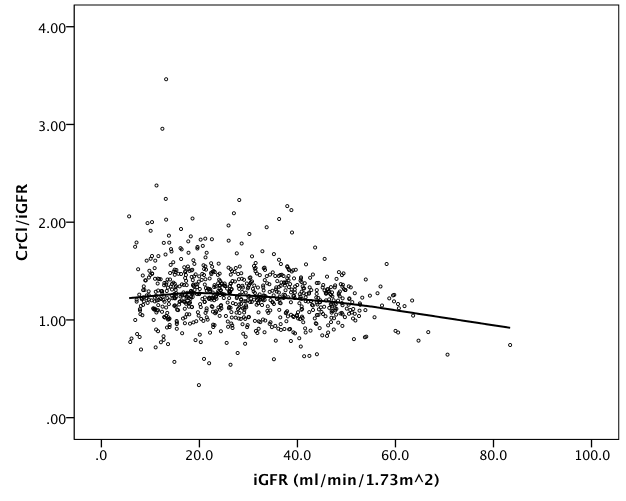 | B.  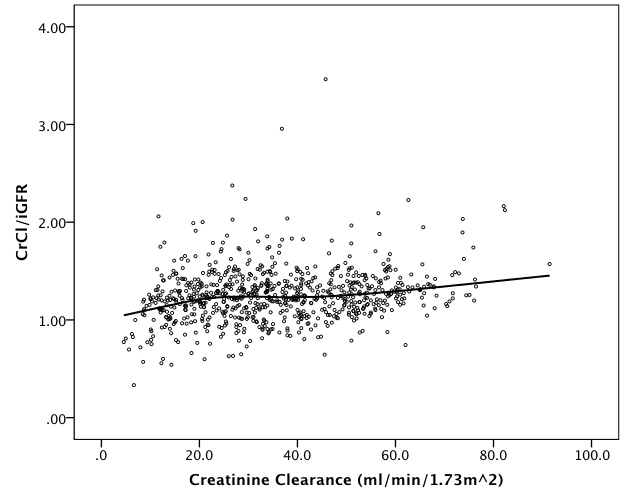 |
| --- | --- |

**Figure S1. (A) Relationship between CrCl/iGFR ratio and iGFR, (B) Relationship between CrCl/iGFR ratio and CrCl** **(The line shown through the scatterplot is from LOESS) (MDRD data, n=797)**

| A.  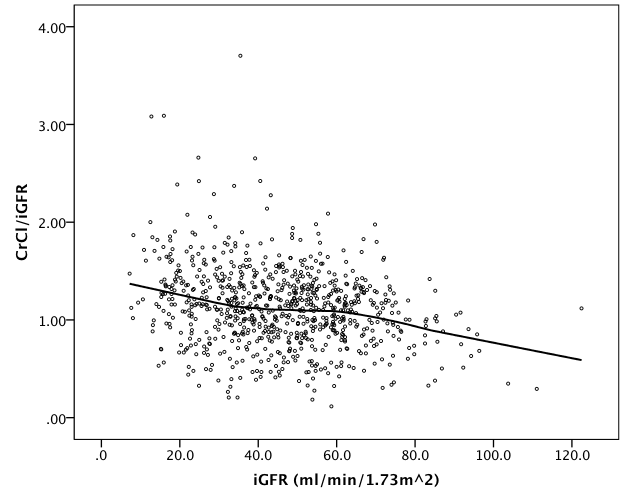 | B.  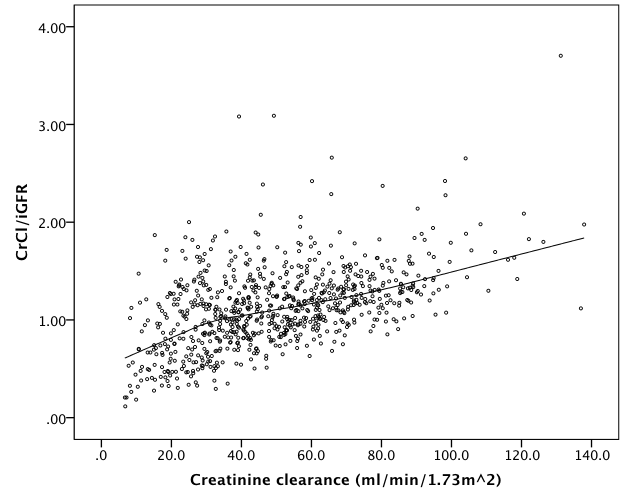 |
| --- | --- |

**Figure S2.** **(A) Relationship between CrCl/iGFR ratio and iGFR, (B) Relationship between CrCl/iGFR ratio and CrCl** **(The line shown through the scatterplot is from LOESS) (AASK data, n=802)**
