# Supplementary material for: Tubular secretion of creatinine and kidney function: an observational study
Source: BMC Nephrol. 2020 Mar 30;21:108. doi: 10.1186/s12882-020-01736-6 (PMC7104490; doi:10.1186/s12882-020-01736-6)
Supplement: Supplementary file 3 — Additional file 3 Fig. S3. Change in mean (±SD) iGFR and mean (±SD) CrCl/iGFR ratio longitudinally among AASK study participants divided into those with decreasing iGFR or increasing iGFR. Fig. S4. Change in mean (±SD) CrCl and mean (±SD) CrCl/iGFR ratio longitudinally among AASK study participants divided into those with decreasing CrCl or increasing CrCl. [file 12882_2020_1736_MOESM3_ESM.docx]

| 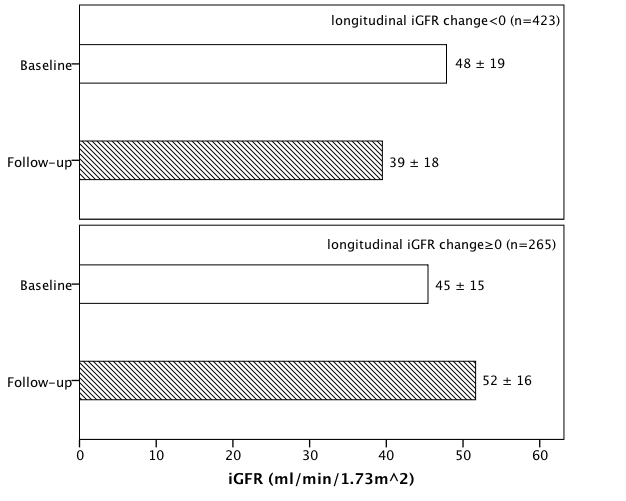 | 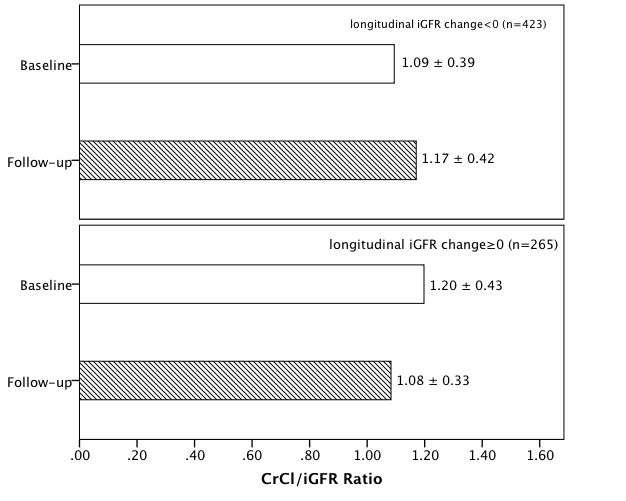 |
| --- | --- |

**Figure S3. Change in mean (±SD) iGFR and mean (±SD) CrCl/iGFR ratio longitudinally among AASK study participants divided into those with decreasing iGFR (n=423) or increasing iGFR (n=265)**

| 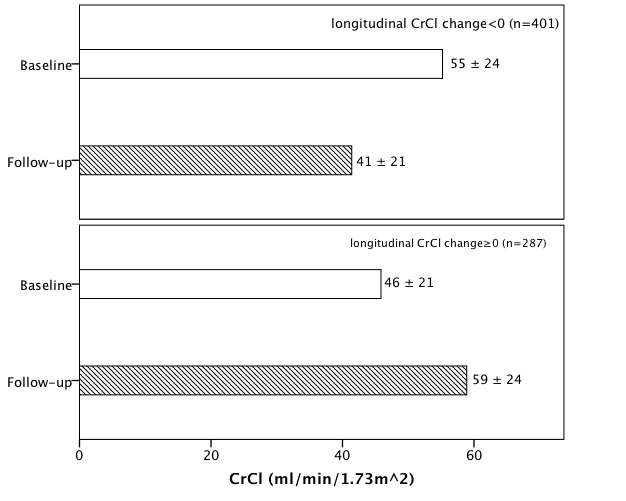 | 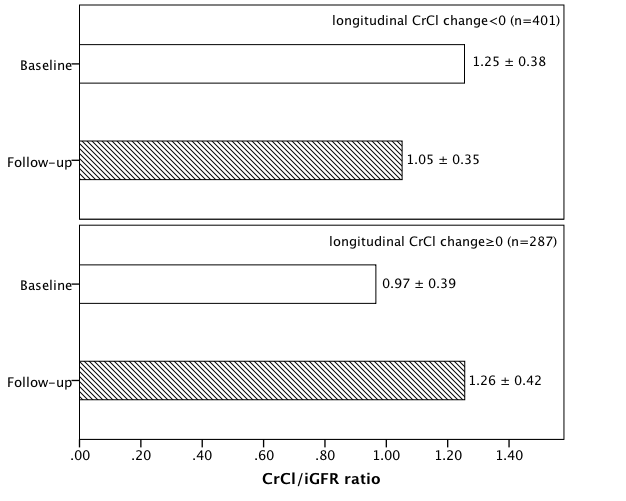 |
| --- | --- |

**Figure S4. Change in mean (±SD) CrCl and mean (±SD) CrCl/iGFR ratio longitudinally among AASK study participants divided into those with decreasing CrCl (n=401) or increasing CrCl (n=287)**
